# Evaluation of potassium clavulanate supplementation of Bolton broth for enrichment and detection of *Campylobacter* from chicken

**DOI:** 10.1371/journal.pone.0205324

**Published:** 2018-10-24

**Authors:** Bai Wei, Min Kang, Hyung-Kwan Jang

**Affiliations:** Department of Veterinary Infectious Diseases and Avian Diseases, College of Veterinary Medicine and Center for Poultry Diseases Control, Chonbuk National University, Iksan, South Korea; University of Campinas, BRAZIL

## Abstract

Culture-based detection of *Campylobacter* can be affected by competing flora, temperature, incubation time, and presence of blood. The presence of extended-spectrum β-lactamase (ESBL)-producing *Escherichia coli* in poultry has become one of the most common factors interfering with the detection of *Campylobacter*. In the present study, we evaluated potassium clavulanate (ESBL inhibitor) as a supplement in Bolton broth (C-Bolton broth) for enrichment and detection of *Campylobacter*. First, we determined growth kinetics of *Campylobacter* in the presence of different concentrations of ESBL *E*. *coli* in C-Bolton broth during enrichment. The effects of other factors such as incubation time, incubation temperature, and presence of blood on *Campylobacter* detection in C-Bolton broth were also investigated. The growth of *Campylobacter* co-cultured at a low concentration (2 and 4 log_10_ CFU/mL) of ESBL *E*. *coli* was similar to that of *Campylobacter* alone in C-Bolton broth, and *Campylobacter* co-cultured at a high concentration (6 and 8 log_10_ CFU/mL) of ESBL *E*. *coli* showed slower growth than the pure *Campylobacter* culture. The *Campylobacter* detection limit was 1 log_10_ CFU/mL when mixed with 2, 4, or 6 log_10_ CFU/mL of *E*. *coli* and 3 log_10_ CFU/mL when mixed with 8 log_10_ CFU/mL of *E*. *coli* after 48 h enrichment in the broth. *Campylobacter* detection from chicken feces and litter samples was not affected by incubation time, or presence of blood in the broth. A modified procedure of enrichment in C-Bolton broth at 37°C for 24 h without blood showed a significantly (*P* ≤ 0.05) higher detection rate and a lower false-negative rate than the ISO 10272–1:2006 method for *Campylobacter* detection from chicken feces and litter samples. In summary, the present study demonstrates the efficacy of Bolton broth supplemented with potassium clavulanate in the detection of *Campylobacter* mixed with ESBL *E*. *coli*, and an improved procedure to detect *Campylobacter* from chicken feces and litter samples.

## Introduction

Campylobacteriosis is a significant and increasing problem to human health both in developed and developing countries. *Campylobacter* is the most common bacterial cause of gastroenteritis with 246,307 confirmed cases of campylobacteriosis in 2016 in Europe [1]. *Campylobacter jejuni* and *C*. *coli* account for over 99% of campylobacteriosis cases [1]. The most common clinical symptoms of *Campylobacter* infections include diarrhea, abdominal pain, fever, headache, nausea, and vomiting. The majority of human campylobacteriosis cases is sporadic, with poultry being the natural reservoir and thought to be the most important vehicle of infection [2].

Effective monitoring and epidemiological investigation of *Campylobacter* requires the use of known techniques and selective medium to ensure proper detection and characterization. Currently, enrichment in Bolton broth at 37°C for 4–6 h followed by incubation for 44–48 h at 41.5°C, and sub-culture in a second selective medium with modified charcoal cefoperazone deoxycholate agar (mCCDA) was recommended by the International Organization for Standardization (ISO) for detection of thermotolerant *Campylobacter* from samples including food, feces, and other environmental samples [3]. Culture-based detection of *Campylobacter* can be affected by competing flora; the presence of extended-spectrum β-lactamase (ESBL)-producing *Escherichia coli* in poultry has become one of the most common factors interfering with *Campylobacter* isolation. Meanwhile, the suppression of *Campylobacter* growth in enrichment media due to overgrowth of ESBL E. *coli* in Bolton broth has been reported [4]. Therefore, elimination of ESBL *E*. *coli* during *Campylobacter* enrichment via addition of an ESBL inhibitor (potassium clavulanate) in Bolton broth has been implemented, and the modified Bolton broth showed more efficient *Campylobacter* recovery from chicken carcasses compared to the unmodified broth [5, 6].

The primary route of human *Campylobacter* transmission is from chicken directly or indirectly along the food production chain, and improvement of intervention strategies that reduce human exposure requires decreasing *Campylobacter* contamination in chicken flock at the source. Both chicken feces and litter are highly contaminated with *Campylobacter* and are important indicators to monitor contamination in chicken [7]. In contrast to carcasses, both feces and litter from farms are contaminated with high levels of competing bacteria such as ESBL *E*. *coli* [8, 9]. Several studies have demonstrated the effectiveness of enrichment in Bolton broth in isolating *Campylobacter* from feces and litter [10, 11]. However, there have been few studies to develop a selective medium for *Campylobacter* isolation from feces and litter. Despite the fact that enrichment in Bolton broth supplemented with potassium clavulanate could inhibit growth of low levels of competing flora and showed an enhanced effect in detecting *Campylobacter* from chicken carcasses [5], its selective power in detecting *Campylobacter* in chicken feces and litter with high levels of the competing flora is unknown. High levels of ESBL *E*. *coli* have been found contaminating in chicken feces and litter, and increasing prevalence of ESBL *E*. *coli* has been reported world-wide along with the fact that β-lactamase antibiotics are still one of the most common antibiotics used in farm animals [1]. However, elimination of different levels of ESBL *E*. *coli* in chicken feces and litter using this modified medium is still not sufficiently studied. In addition to competitive inhibition, *Campylobacter* isolation may also be affected by factors including the presence of blood in the selective medium, incubation time, and incubation temperature, because *Campylobacter* is very sensitive to environmental stress including variations in temperature, pH, and atmospheric oxygen [12–14]. To improve the *Campylobacter* detection method, the first aim of the present study was to determine the growth kinetics of *Campylobacter* mixed with different concentrations of ESBL *E*. *coli* in Bolton broth supplied with potassium clavulanate during enrichment. In addition, to adapt the ISO 10272–1:2006 procedure for *Campylobacter* detection from chicken feces and litter samples, the effect of modification of factors including incubation temperature, incubation time, and presence of blood on *Campylobacter* detection in the broth were also investigated.

## Materials and methods

### Bacterial strains and preparation of stationary phase cultures

The reference strain *C*. *jejuni* ATCC 33560 and a field strain of *C*. *coli* isolated from chicken meat were used to assess the growth kinetics. One ESBL-producing *E*. *coli* strain carrying the *CTX-M-15* gene (Accession No. MH756636) was isolated from chicken meat previously in our lab and the gene was confirmed via sequencing. The *Campylobacter* strains were maintained at −70°C in Bolton broth (Oxoid Ltd., Basingstoke, England) with 10% horse blood (Oxoid) and 20% glycerol, and the stock was plated on 5% sheep blood agar plates (Komed, Seongnam, South Korea) at 41.5°C for 48 h under microaerophilic condition (10% CO_2_, 5% O_2_, and 85% N_2_). The *E*. *coli* stock was maintained in Tryptone Soy broth (Difco, Maryland, USA) and plated on MacConkey agar (Difco, Maryland, USA). Subsequently, single colonies were suspended in MH broth (Becton, Maryland, USA) to obtain stationary phase cultures, and bacterial concentrations of *Campylobacter* and *E*. *coli* were determined via serial dilution and plating on blood agar with 48 h incubation and MacConkey agar with 24 h incubation, respectively.

### Media preparation

Bolton broth was prepared following the manufacturer’s instruction. It contained 20 mg/L cefoperazone, 20 mg/L vancomycin, 20 mg/L trimethoprim lactate, 10 mg/L amphotericin B, and 5% lysed defibrinated horse blood. C-Bolton broth was prepared using Bolton broth supplemented with potassium clavulanate (Sigma-Aldrich, St. Louis, MO, USA) to a final concentration of 2 mg/L [5]. Modified charcoal cefoperazone deoxycholate agar (mCCDA, Oxoid) was prepared per the manufacturer’s recommendations using mCCDA antibiotic supplement (32 mg/L cefoperazone and 10 mg/L amphotericin; Oxoid). C-mCCDA was generated by adding 0.5 mg of potassium clavulanate to 1 L of cooled mCCDA [15].

### Measuring growth dynamics

The growth dynamics of *Campylobacter* and ESBL *E*. *coli* in MH broth were established. The growth of *Campylobacter* and ESBL *E*. *coli* co-culture in Bolton broth was determined at 41.5°C for 48 h. After enrichment, broth samples were immediately diluted and plated on agar plates to determine the colony forming units (CFUs). The CFUs of ESBL *E*. *coli* were determined on MacConkey agar. The broth was serially diluted and plated on C-mCCDA, which also inhibit ESBL *E*. *coli* growth to count *Campylobacter*. All the *Campylobacter* morphological colonies were counted on C-mCCDA, and the atypical colonies were not included in counting.

Next, the growth dynamics of *C*. *jejuni* and *C*. *coli* were determined in C-Bolton broth at initial concentrations of 1–3 log_10_ CFU/mL with incubation for 48 h. Different combinations of *Campylobacter* (at 1, 2, or 3 log_10_ CFU/mL) with *E*. *coli* at 2, 4, 6, or 8 log_10_ CFU/mL were used to establish the growth dynamics of *Campylobacter* in C-Bolton broth. Each experiment was carried out in duplicate to determine the growth dynamics, and the *Campylobacter* CFU values were determined at time points of 3, 6, 12, 24, and 48 h.

### Chicken feces and litter sample collection

Eight broiler chicken farms of a company in South Korea were sampled. A total of 40 pooled feces and 24 pooled litter samples were collected from the eight farms located in Chonbuk province. Five pooled fresh feces samples were randomly obtained from a whole flock in one farm. The flock was divided equally into three zones for sampling, and three pooled broiler litter samples were collected randomly from each zone. All samples were transported to the laboratory in insulated boxes with ice packs and were processed immediately upon arrival. A trained veterinarian collected the feces and litter from the environment of the chicken farms, the owners of each farm provided permission for sample collection, and no ethical approval was required for the study because the procedures did not involve animal handling and caused no harm to them.

### Detection of *Campylobacter* spp. in chicken feces and litter samples

Two methods (direct plating onto agar and enrichment culturing) were used for all feces and litter samples. First, direct plating was performed per the ISO 10272–2:2006 procedures for *Campylobacter* detection [16]. One gram of each feces or litter sample was suspended in 9 mL of Buffered Peptone Water (BPW; BD Difco, Sparks, MD, USA) and mixed thoroughly, and then each sample was individually inoculated onto mCCDA and C-mCCDA plates. All plates were incubated at 41.5°C for 48 h in a microaerophilic environment. Second, enrichment was performed per the ISO 10272–1:2006 procedures for *Campylobacter* detection. A 5-mL volume of each sample was mixed with an identical volume of 2X Bolton broth or 2X C-Bolton broth. Each sample was incubated at 37°C for 4 h, and then transferred to 41.5°C in a microaerophilic environment. Following incubation in Bolton or C-Bolton broth for 48 h, each enriched sample was streaked onto mCCDA followed by incubation at 41.5°C in a microaerophilic environment for 48 h.

To determine the effect of incubation temperature, incubation time, and blood on *Campylobacter* detection in C-Bolton broth, all feces and litter samples were also enriched in C-Bolton broth with or without the addition of lysed defibrinated horse blood. Each sample was incubated at 37°C for 4 h, and then transferred to 37°C or 41.5°C for 24 h or 48 h in a microaerophilic environment. Following incubation in C-Bolton broth for 24 h or 48 h, each enriched sample was streaked onto mCCDA followed by incubation at 41.5°C in a microaerophilic atmosphere for 48 h.

Presumptive *Campylobacter* colonies on the mCCDA plates were further cultivated on 5% sheep blood agar plates at 41.5°C for 24–48 h under microaerophilic conditions. Isolates were identified to the genus level by amplifying the *16S rRNA* gene using primers specific to *Campylobacter* species [17].

### Data analysis

The positive results obtained in detection using different enrichment/plating combinations were cross-checked and a sample was assigned to be positive based on the use of any one procedure that was successful in isolating *Campylobacter*. The corresponding procedures yielding negative results for the same sample were considered false-negative. The false-negative rate was calculated using the formula 100 * (False Negative) / (True Positive + False Negative) [18]. Differences in *Campylobacter* detection from chicken feces and litter between direct plating on mCCDA and C-mCCDA, and between enrichment in Bolton and C-Bolton broth, were compared using the Chi-Square test and the software SPSS version 19.0 (IBM, Armonk, NY, USA). Significant differences in detection and false-negative rate via enrichment in C-Bolton broth due to enrichment time, temperature, or presence of blood were also analyzed using the Chi-Square test. Further, every modified procedure was compared with the standard ISO 10272–1:2006 procedure using Bolton broth supplemented with potassium clavulanate. The threshold for statistical significance was set at *P* ≤ 0.05.

## Results

### Growth dynamics of pure or mixed cultures

**Fig 1:**
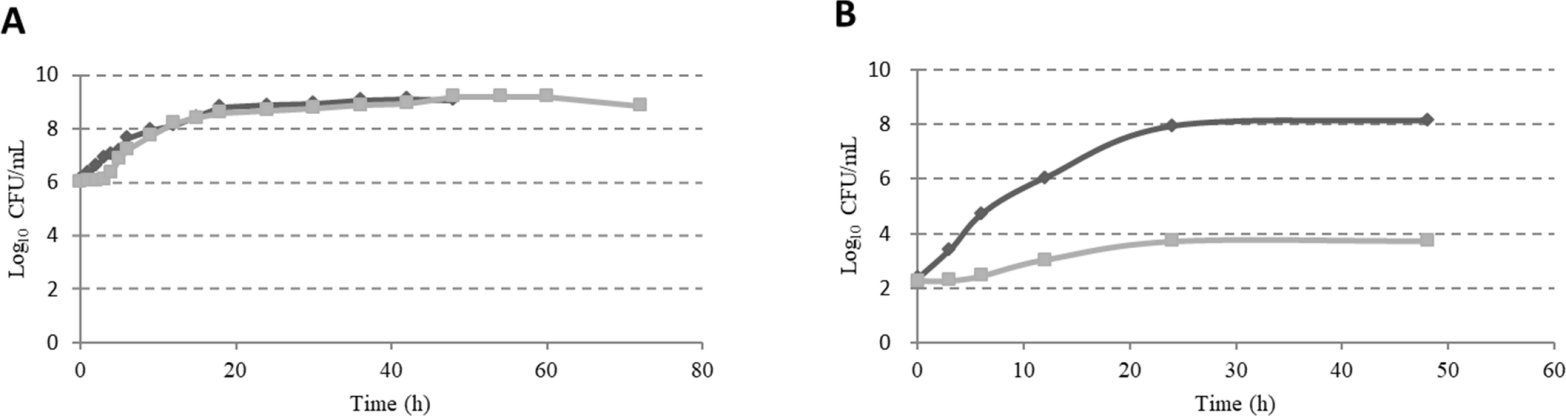
Growth of *Campylobacter* and ESBL *E*. *coli* in MH broth and Bolton broth. Growth curve of *Campylobacter* (square) cultured for 72 h and of ESBL *E*. *coli* (rhombus) cultured for 48 h in MH broth when cultured separately (A). Growth of *Campylobacter* and ESBL *E*. *coli* in Bolton broth when co-cultured at 41.5°C under microaerophilic conditions (B).

Growth curves of *Campylobacter* cultured for 72 h and of ESBL *E*. *coli* cultured for 48 h in MH broth are showed in Fig 1A, when cultured separately. Both *Campylobacter* and ESBL *E*. *coli* peaked at approximately 9 log_10_ CFU/mL in MH broth at 30 and 18 h, respectively. The growth curves of *Campylobacter* and ESBL *E*. *coli* cultured together in Bolton broth are shown in Fig 1B. A decrease in the number of *Campylobacter* with the incubation time was noted, and less than 4 log_10_ CFU/mL of *Campylobacter* was present in Bolton broth after 48 h enrichment.

**Fig 2:**
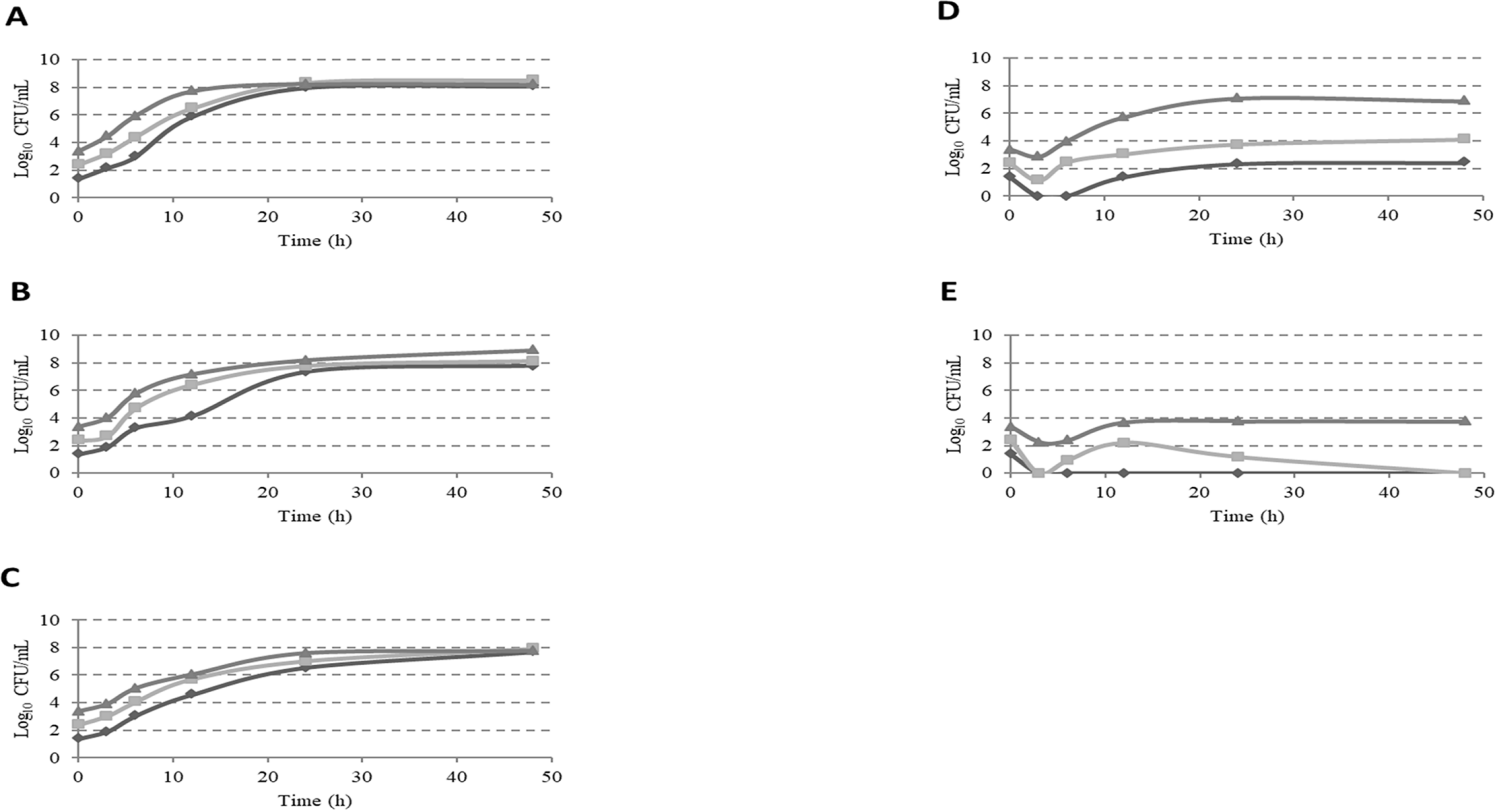
Growth curve of *Campylobacter* in C-Bolton broth with or without ESBL-producing *E*. *coli*. *Campylobacter* was cultured alone in C-Bolton broth for 48 h at initial concentrations of 1 (rhombus), 2 (square), or 3 (triangle) log_10_ CFU/mL (A). Mixed cultures of *Campylobacter* at concentrations of 1–3 log_10_ CFU/mL with *E*. *coli* at 2 log_10_ CFU/mL (B), 4 log_10_ CFU/mL (C), 6 log_10_ CFU/mL (D), or 8 log_10_ CFU/mL in C-Bolton broth (E). The detection limit was 10 CFU/mL, and *Campylobacter* concentration was quantified at 3, 6, 12, 24, and 48 h.

Using a starting inoculum of 1–3 log_10_ CFU/mL, growth dynamics of pure *C*. *jejuni* and *C*. *coli* strains in C-Bolton broth are shown in Fig 2A. Maximum concentrations of 8 log_10_ CFU/mL were attained after culturing for approximately 24 h, with the same concentrations found at 48 h. All experiments for evaluating growth dynamics in mixed culture involved co-culture with *C*. *jejuni* and *C*. *coli* or ESBL *E*. *coli*, because the *C*. *jejuni* and *C*. *coli* strains showed similar results in terms of average growth rates.

Fig 2B and 2C show the growth kinetics of *Campylobacter* in combination with a low concentration of 2 or 4 log_10_ CFU/mL of ESBL *E*. *coli* in C-Bolton broth. It can be noticed that the growth curve of *Campylobacter* was the same as that in pure culture; the peak concentration of *Campylobacter* was attained after approximately 24 h of culture and there was no obvious trend of increase in CFU values after 24–48 h of enrichment.

Fig 2D and 2E show the growth kinetics of *Campylobacter* in combination with a high concentration of 6 or 8 log_10_ CFU/mL of *E*. *coli* in C-Bolton broth. An obvious decreasing trend in *Campylobacter* concentrations with all three starting inoculums (1, 2, or 3 log_10_ CFU/mL) was obtained at 3 h after enrichment in C-Bolton broth with *E*. *coli*. All three inoculums also showed a significantly lower number of *Campylobacter* at 48h compared with the pure culture. The starting inoculums at 1, 2, and 3 log_10_ CFU/mL mixed with *E*. *coli* at 6 log_10_ CFU/mL resulted in 5.65, 4.39, and 1.31 log_10_ CFU/mL reductions in *Campylobacter* respectively, over the pure *Campylobacter* culture after 48 h of enrichment (Fig 2D). The starting inoculums at 1 and 2 log_10_ CFU/mL mixed with *E*. *coli* of at 8 log_10_ CFU/mL resulted in no recovery of *Campylobacter* after 48 h of enrichment in C-Bolton broth (Fig 2E). *Campylobacter* at 3 log_10_ CFU/mL mixed with *E*. *coli* at 8 log_10_ CFU/mL resulted in a 4.45 log_10_ CFU/mL reduction in *Campylobacter* over the pure *Campylobacter* culture after 48 h of enrichment.

### *Campylobacter* recovery using direct plating or enrichment

*Campylobacter* recovery from selective media was investigated by directly plating 40 feces and 24 litter samples collected from the chicken farms (Table 1). Direct plating onto C-mCCDA (64.1%, 41/64) resulted in higher (*P* ≤ 0.05) number of *Campylobacter* compared to direct plating onto mCCDA (46.9%, 30/64). A higher (*P* ≤ 0.05) number of *Campylobacter* was also recovered after enriching in C-Bolton broth (68.8%, 44/64) compared to that in Bolton broth (51.6%, 33/64).

**Table 1:** Detection of *Campylobacter* in chicken feces and litter samples (%) using direct plating or enrichment.

Sample type	Positive samples (%)			
	Direct plating		Enrichment	
	mCCDA	C-mCCDA	Bolton	C-Bolton
**Feces (n = 40)**	25 (62.5)	30 (75.0)	25 (62.5)	31 (77.5)
**Litter (n = 24)**	5 (20.8)	11 (45.8)	8 (33.3)	13 (54.2)
**Total (n = 64)**	30 (46.9)^a^	41 (64.1)^b^	33 (51.6)^c^	44 (68.8)^d^

C-mCCDA/C-Bolton: potassium clavulanate in mCCDA/Bolton.

The statistical comparison was performed between the results of direct plating on mCCDA and C- mCCDA, enrichment in Bolton and C-Bolton broth (c / d), separately. Different superscripts (^a/b^ and ^c/d^) indicate statistically significant differences (*P* ≤ 0.05).

### Evaluation of enrichment conditions using C-Bolton broth for *Campylobacter* detection in chicken feces and litter samples

*Campylobacter* detection in chicken feces and litter samples with enrichment in C-Bolton broth under different culture conditions was evaluated and the detection results are summarized in Table 2. Regardless of the incubation time, temperature, or presence of blood in the broth, *Campylobacter* detection rates in chicken feces or litter samples showed no significant differences (*P* > 0.05).

**Table 2:** Detection of *Campylobacter* in chicken feces and litter samples (%) via enrichment in C-Bolton broth with different culture conditions.

Sample type	5% blood	Positive samples (%)			
		Incubation time (h) at 37°C		Incubation time (h) at 41.5°C	
		24	48	24	48
**Feces (n = 40)**	With	33 (82.5)	31 (77.5)	32 (80.0)	31 (77.5)*
	Without	37 (92.5)	34 (85.0)	32 (80.0)	32 (80.0)
**Litter (n = 24)**	With	15 (62.5)	13 (54.2)	13 (54.2)	13 (54.2)*
	Without	19 (79.2)	16 (66.7)	14 (58.3)	15 (62.5)
**Total (n = 64)**	With	48 (75.0)	44 (68.8)	45 (70.3)	44 (68.8)*^b^
	Without	56 (87.5)^a^	50 (78.1)	46 (71.9)	47 (73.4)

*, the ISO method with potassium clavulanate supplementation.

The statistical comparison was performed between the results of enrichment in C-Bolton broth for 24 and 48 h, at 37 and 41.5°C, with and without 5% blood. Furthermore, every modified procedure was compared with the standard ISO 10272–1:2006 procedure using Bolton broth supplemented with potassium clavulanate. Different superscripts (^a/b^) indicate statistically significant differences (*P* ≤ 0.05).

The overall *Campylobacter* detection rates from chicken feces and litter samples ranged from 68.8% to 87.5% under different conditions (Table 2). The detection rate using the ISO 10272–1:2006 procedure with potassium clavulanate supplementation was 77.5% (31/40) and 54.2% (13/24) for feces and litter samples, respectively. Among all the enrichment procedures, a procedure using enrichment in C-Bolton broth with incubation at 37°C for 24 h and absence of blood showed the highest detection rate (87.5%) which was significantly higher (*P* ≤ 0.05) than that using the ISO 10272–1:2006 procedure.

### Effect of enrichment conditions on false-negative detection of *Campylobacter* in chicken feces and litter samples

The false negative detection rates using different enrichment conditions for *Campylobacter* detection from chicken feces and litter samples are summarized in Table 3. The procedure involving enrichment in C-Bolton broth with incubation at 37°C for 24 h and absence of blood showed a lower (*P* ≤ 0.05) false negative rate (1.8%) than the procedure with incubation at 41.5°C for 24 h and absence of blood (19.3%) and the procedure with incubation at 37°C for 24 h with blood (15.8%). There were no significant differences (*P* > 0.05) in the false negative detection rates from feces or litter samples to changes in any other factors including incubation time, temperature, and presence of blood in the broth.

**Table 3:** False-negative detection of *Campylobacter* in chicken feces and litter samples (%) via enrichment in C-Bolton under different culture conditions.

Sample type	5% blood	False-negative samples (%)			
		Incubation time (h) at 37°C		Incubation time (h) at 41.5°C	
		24	48	24	48
**Feces (n = 37)**	With	4 (10.8)	6 (16.2)	5 (13.5)	6 (16.2)*
	Without	0	3 (8.1)	5 (13.5)	3 (8.1)
**Litter (n = 20)**	With	5 (25.0)	7 (35.0)	7 (35.0)	7 (35.0)*
	Without	1 (5.0)	4 (20.0)	6 (30.0)	5 (25.0)
**Total (n = 57)**	With	9 (15.8)^d^	13 (22.8)	12 (21.1)	13 (22.8)*^f^
	Without	1 (1.8)^a, c, e^	7 (12.3)	11 (19.3)^b^	8 (14.0)

*, the ISO method with potassium clavulanate supplementation.

The statistical comparison was performed between the false-negative results of enrichment in C-Bolton broth for 24 and 48 h, at 37 and 41.5°C, with and without 5% blood. Furthermore, every modified procedure was compared with the standard ISO 10272–1:2006 procedure using Bolton broth supplemented with potassium clavulanate. Different superscripts (^a/b^, ^c/d^ and ^e/f^) indicate statistically significant differences (*P* ≤ 0.05).

*Campylobacter* recovery after enrichment in C-Bolton broth showed differences in false negative findings under different enrichment conditions. The false-negative rate varied from 0 to 6 (16.2%) in feces samples and from 1 (5.0%) to 7 (35.0%) in litter samples. The false negative rate using the ISO 10272–1:2006 procedure supplemented with potassium clavulanate was 16.2% (6/37) and 35.0% (7/20) in feces and litter samples, respectively. Among all the enrichment procedures, a procedure involving enrichment in C-Bolton broth with incubation at 37°C for 24 h and absence of blood showed the lowest false negative rate (1.8%), which was significantly lower (*P* ≤ 0.05) than that using the ISO 10272–1:2006 procedure.

## Discussion

Although various selective agar media and enrichment broths have been developed and introduced for the isolation of *Campylobacter*, the Bolton broth is the most common medium used to detect *Campylobacter* spp. [14]. However, the increasing prevalence of ESBL *E*. *coli* has become the primary factor hindering the detection of *Campylobacter* in Bolton broth [4]. Therefore, inhibiting ESBL *E*. *coli* using an ESBL inhibitor has attracted attention and has shown significant benefits in detecting *Campylobacter* in chicken products [5]. A previous study showed that *Campylobacter* growth was not competitively inhibited due to a low level of ESBL *E*. *coli* in Bolton broth supplemented with potassium clavulanate in artificial and natural samples [6]. However, the efficiency of this modified medium in detecting *Campylobacter* from environmental samples with high levels of competitive bacteria including ESBL *E*. *coli* has not been determined. Therefore, we conducted the present study to investigate the growth kinetics of *Campylobacter* in C-Bolton broth with competitive ESBL *E*. *coli*. *Campylobacter* growth has been shown to be inhibited in Bolton broth at concentrations < 3 log_10_ CFU/mL in the presence of competitive ESBL *E*. *coli*, with no *Campylobacter* recovery from the second agar plate [4]. In the present study, we used 1, 2, or 3 log_10_ CFU/mL of *Campylobacter* mixed with different concentrations of competitive ESBL *E*. *coli*.

Our results showed that the growth of *Campylobacter* mixed with 2 or 4 log_10_ CFU/mL of ESBL *E*. *coli* was similar to that of *Campylobacter* alone in C-Bolton broth, and the total number of *Campylobacter* after incubation for 48 h were also similar to those using the pure culture. Our findings are in agreement with those of a previous study which showed that low ESBL *E*. *coli* contamination could be inhibited quickly and did not affect *Campylobacter* growth in C-Bolton broth [6]. In contrast, the total number of *Campylobacter* in mixed cultures with 6 or 8 log_10_ of CFU/mL *E*. *coli* in C-Bolton broth was at least 2 log_10_ CFU/mL lower than that in the pure culture after incubation for 48 h. In addition, the number of *Campylobacter* in the above cultures was lower at every time point than that in the pure culture of *Campylobacter* or in the mixed cultures of *Campylobacter* with low levels of ESBL *E*. *coli*. The low numbers of *Campylobacter* at every point suggest that ESBL *E*. *coli* existed persistently in the broth. This may have been because the concentrations of β-lactams (cefoperazone) and/or the β-lactamase inhibitor (potassium clavulanate) in the broth were not sufficient to inhibit high concentrations of ESBL *E*. *coli*. This observation is also supported by the fact that the effects of β-lactams and β-lactamase inhibitors are reduced in the presence of high β-lactamase production [19, 20]. According to a previous study, Bolton broth supplemented with 2 mg/L of potassium clavulanate did not inhibit the growth of *Campylobacter* and showed a detection rate higher than Bolton broth with 10 mg/L [5]; this concentration was used in the present study. Because 10 mg/L not only inhibited the contamination but also some *Campylobacter*, concentrations between 2 and 10 mg/L potassium clavulanate in the broth may require further optimization to improve selectivity. Further, the concentration of potassium clavulanate between 2 and 10 mg/L may not fully inhibit β-lactamase production. Alternatively, additional potassium clavulanate may have to be added to the broth after several hours of incubation to fully inhibit β-lactamase production; a previous report showed that an additional β-lactamase inhibitor during enrichment caused a decrease in regrowth of ESBL bacterial [19]. Using another β-lactamase inhibitor such as tazobactam, which has been used in *Campylobacter* detection [21], may be an alternative choice.

A decrease in *Campylobacter* number was seen in the broth at 3 h compared with the initial inoculum when mixed with 6 or 8 log_10_ CFU/mL of ESBL *E*. *coli* (Fig 2D and 2E). A decrease in *Campylobacter* during enrichment in the selective medium Preston broth which also inhibits the growth of ESBL *E*. *coli* has been reported previously in mixed cultures with low concentrations of ESBL *E*. *coli* [4]. This is supported by the observation that *Campylobacter* growth is suppressed easily by ESBL bacteria even in an enrichment medium in which ESBL *E*. *coli* could not grow [4]. It is noteworthy that the number of *Campylobacter* increased after 3 h and declined again after an incubation of 12 h when 2 log_10_ CFU/mL of *Campylobacter* were co-cultured with 8 log_10_ CFU/mL of ESBL *E*. *coli* (Fig 2E). This suggests that the high concentration of ESBL *E*. *coli* was primary inhibited by the β-lactam and β-lactamase inhibitor in the C-Bolton broth, resulting in *Campylobacter* proliferation. A decrease in the effect of the β-lactam and β-lactamase inhibitor in the broth over time may have led to regrowth of incompletely inhibited ESBL *E*. *coli*, resulting in suppression of *Campylobacter* again. Following the continued growth of ESBL *E*. *coli*, the numbers of *Campylobacter* decreased to levels below the detection limit from 12 h to 48 h. The basis for decreased *Campylobacter* enrichment in C-Bolton broth in the presence of high ESBL *E*. *coli* concentrations is unclear. The strain of ESBL *E*. *coli* used may have the role to competitively inhibit the growth of other bacteria [22], and the ratio of *Campylobacter* and ESBL *E*. *coli* may have affected the growth of *Campylobacter*. In addition, the *E*. *coli* may express molecules killing competing bacteria including antimicrobial peptides, type VI secretion systems, or toxic proteins [23, 24]. Further studies are required to test these hypotheses and reach conclusions.

In our study, the *Campylobacter* detection limit was 1 log_10_ CFU/mL when mixed with 2, 4, or 6 CFU/mL of *E*. *coli* and 3 log_10_ CFU/mL when mixed with 8 log_10_ CFU/mL of *E*. *coli* after 48 h enrichment in C-Bolton broth. Jasson et al., [4] showed that *Campylobacter* could not be recovered after enrichment in Bolton broth mixed with the same number (approximately 2 log_10_ CFU/mL) of ESBL *E*. *coli*, and there have been no reports regarding the detection limit of *Campylobacter* in C-Bolton broth. Our study extends previous ones to evaluate *Campylobacter* recovery when mixed with low or high levels of contaminating competing ESBL bacteria in C-Bolton broth. We found that the *Campylobacter* detection limit was 2 log_10_ CFU/mL when mixed with 8 log_10_ CFU/mL of *E*. *coli* after 24 h enrichment in C-Bolton broth, with no obvious increase in the number of *Campylobacter* comparing 24 h and 48 h enrichment in pure or various mixed cultures. Considering these results, a decreased enrichment time is likely to be beneficial in detecting *Campylobacter* from samples with low or high competing bacteria [10].

Both direct plating and enrichment are useful in detecting *Campylobacter* from food-related samples [11, 14]. However, few studies have focused on environmental samples and there has been no widely accepted protocol for the detection of *Campylobacter* from such samples. In contrast to a previous study, which showed that direct plating had higher selectivity than enrichment from chicken feces and litter [10], the present study demonstrated that both direct plating onto C-mCCDA or enrichment in C-Bolton broth were effective in improving the detection of *Campylobacter* from chicken feces and litter samples. This suggests that both direct plating and enrichment are useful in detecting *Campylobacter* [5, 15]. After inhibition of low or high levels of competing bacteria, the enrichment method showed a higher detection rate in the present study, suggesting that enrichment in a selective medium is likely to be advantageous over the direct plating method [25].

Our results also highlighted an improved procedure for *Campylobacter* isolation from chicken feces and litter samples. Enrichment in C-Bolton broth at 37°C for 24 h in the absence of blood showed a higher *Campylobacter* detection rate from chicken feces or litter than the ISO 10272–1:2006 procedure. Further, the modified procedure also showed a significantly higher (*p* ≤ 0.05) detection rate (87.5%) from both chicken feces and litter when the samples were analyzed together (Table 2). This suggests that a reduced enrichment time (from 48 h to 24 h), saved labor (no need to move samples from 37°C to 41.5°C) and lower cost (no need for blood) would result in a higher detection rate from chicken samples. Early studies have shown that an extended incubation time induces a higher number of *Campylobacter* with slow growth characteristics, a higher temperature provides competitive benefit to *Campylobacter* growth, and supplementation with blood reduces damage caused by oxidative toxins in Bolton broth [26, 27]. However, an increasing number of studies have confirmed that the above modifications do not improve *Campylobacter* detection from chicken samples. Williams et al., [14] found no difference between incubation temperatures of 37°C and 41.5°C, and between incubation times of 24 h and 48 h for *Campylobacter* detection. Several studies showed that blood supplementation in Bolton broth had no effect on the recovery rates [28, 29]. In addition, these modifications have been shown to decrease detection rates in several reports. Vaz et al., [10] found a significantly lower *Campylobacter* detection after 48 h enrichment in Bolton broth. A lower number of *Campylobacter* was recovered after enrichment in Bolton broth in the presence of blood, and the USDA Food Safety and Inspection Service has recommended the use of blood-free Bolton broth to isolate *Campylobacter* from chicken [30, 31]. The present study showed that there was no significant difference due to the modifications, even in the C-Bolton broth. Minor variations, low sample number, or other unknown reasons may underlie our results. Further studies are required to address the possible causes.

Failure to detect foodborne pathogens increases the risk of their transmission. *Campylobacter* spp. is a particularly fastidious microorganism and is the most common false-negative foodborne pathogen in the USA with an average false negative rate of 13.6% from 1999 to 2007 [32]. In the present study, all evaluation procedures gave false negative results in *Campylobacter* detection, and the ISO 10272–1:2006 procedure showed the maximum false negative rate. The best procedure for both feces and litter samples was sample enrichment in C-Bolton broth at 37°C for 24 h in the absence of blood, with one false-negative sample from litter and none from feces. Further, this procedure showed a significantly lower (*P* ≤ 0.05) false-negative rate (1.8%) than the ISO 10272–1:2006 procedure (22.8%) in the feces and litter samples. The modified procedure also showed a lower false negative rate than procedures involving temperature change from 41.5°C to 37°C or the presence of blood. Although lower temperature and absence of blood have not been reported to have a direct effect on decreasing false negative rates, Khan et al. [33] reported that some *Campylobacter* spp. could not be recovered via enrichment at 42°C and significantly lower numbers of *Campylobacter* were obtained at 42°C from environmental samples. Blood supplementation in the enrichment broth may also benefit other competitive bacteria and result in more contaminants [34]. Therefore, our findings show that cautious selection of the enrichment broth and the enrichment procedure are important in reducing misdetection of *Campylobacter* from chicken samples. We found that all the enrichment procedures gave false-negative results with *Campylobacter* colonies being recovered from mCCDA. Considering that the mCCDA was supplemented with the same antibiotic (cefoperazone) as the Bolton broth, selectivity enhancement in these two selective media is limited. Further investigations are required to modify this procedure for improving *Campylobacter* detection from field samples.

In conclusion, the present study characterizes growth kinetics of *Campylobacter* mixed with different concentrations of ESBL *E*. *coli* in C-Bolton broth during enrichment. The growth of *Campylobacter* was not affected due to mixing with a low concentration of ESBL *E*. *coli* but was partially or completely inhibited due to a high concentration of ESBL *E*. *coli* in the broth. The study also adds to current knowledge regarding the effect of potassium clavulanate in Bolton broth on *Campylobacter* detection from chicken feces and litter along with the effects of temperature, incubation time, and blood. Our results highlight an improved procedure, enrichment in C-Bolton broth at 37°C for 24 h without blood, which showed a higher detection rate and a lower false negative rate than the present ISO 10272–1:2006 method for detection from chicken feces or litter. Our results confirm the need to adapt the ISO 10272–1:2006 method for *Campylobacter* detection from environment samples or other samples with high background flora. Therefore, a modified protocol involving enrichment in C-Bolton broth at 37°C for 24 h without blood is recommended to increase the probability of *Campylobacter* detection saving time, labor, and cost. This procedure may help in improving *Campylobacter* monitoring programs to reduce the number of false negative flocks which would be crucial in controlling *Campylobacter* transmission in the environment. Our study also had several limitations. First, the growth dynamics of ESBL *E*. *coli* in Bolton broth supplement with potassium clavulanate was not determined. Second, the Bolton broth was supplemented with 2 mg/mL of potassium clavulanate and the most effective concentration inhibiting ESBL *E*. *coli* with no effect of *Campylobacter* growth requires further investigation. Third, only one strain of ESBL *E*. *coli* containing the most frequently studied ESBL gene *CTX-M-15* was used and the effect of other β-lactamase resistance genes was not determined. Finally, we did not determine the prevalence of ESBL *E*. *coli* in chicken feces and litter, and the effect of the modified medium on suppression of ESBL bacterial growth in the field is unclear at present. Further studies are necessary to fully understand the influence of different factors and to optimize the detection procedure.
